# Feature context-dependency and complexity-reduction in probability landscapes for integrative genomics

**DOI:** 10.1186/1742-4682-5-21

**Published:** 2008-09-10

**Authors:** Annick Lesne, Arndt Benecke

**Affiliations:** 1 Institut des Hautes Études Scientifiques, Bures-sur-Yvette, France; 2 Institut de Recherche Interdisciplinaire – CNRS USR3078 – Université Lille I, France

## Abstract

**Background:**

The question of how to integrate heterogeneous sources of biological information into a coherent framework that allows the gene regulatory code in eukaryotes to be systematically investigated is one of the major challenges faced by systems biology. Probability landscapes, which include as reference set the probabilistic representation of the genomic sequence, have been proposed as a possible approach to the systematic discovery and analysis of correlations amongst initially heterogeneous and un-relatable descriptions and genome-wide measurements. Much of the available experimental sequence and genome activity information is *de facto*, but not necessarily obviously, context dependent. Furthermore, the context dependency of the relevant information is itself dependent on the biological question addressed. It is hence necessary to develop a systematic way of discovering the context-dependency of functional genomics information in a flexible, question-dependent manner.

**Results:**

We demonstrate here how feature context-dependency can be systematically investigated using probability landscapes. Furthermore, we show how different feature probability profiles can be conditionally collapsed to reduce the computational and formal, mathematical complexity of probability landscapes. Interestingly, the possibility of complexity reduction can be linked directly to the analysis of context-dependency.

**Conclusion:**

These two advances in our understanding of the properties of probability landscapes not only simplify subsequent cross-correlation analysis in hypothesis-driven model building and testing, but also provide additional insights into the biological gene regulatory problems studied. Furthermore, insights into the nature of individual features and a classification of features according to their minimal context-dependency are achieved. The formal structure proposed contributes to a concrete and tangible basis for attempting to formulate novel mathematical structures for describing gene regulation in eukaryotes on a genome-wide scale.

## Background

The deciphering of the gene regulatory code of eukaryotic cells and the inference of gene regulatory programs belong to the computationally "hard" problems that are very probably insoluble without using very large collections of experimental genome activity recordings under many different biological conditions in conjunction with empirical gene structure and function annotations [1-4]. Genomic sequence, gene structure and function annotation, as well as functional genomics experimental data, are of heterogeneous nature. In order to conceive computationally efficient algorithms capable of statistical integration of these different types of information, transformations of the different types of data into a continuous and homogeneous data structure have to be developed. We have recently proposed such a concept, which we refer to as probability landscapes [5]. Briefly, we have shown on theoretical grounds how any type of observable quantity (which we shall refer to hereafter as "feature") can, without loss of information, be transformed into a local probability with nucleotide resolution along the genome (creating what we define as a probability profile). For any feature, as for instance the predicted alpha-helicity of an inferred amino-acid sequence or the transcriptome of a cell recorded under a particular biological condition, such a local probability can be calculated for all nucleotides of the genome under study, resulting in a profile. If this procedure is repeated for many different features, a stack of probability profiles ("landscape") is obtained. While it might, on first sight, seem awkward to calculate a probability for every nucleotide in a genome to be part of an alpha-helix provided this nucleotide were part of an expressed codon, the advantage of translating any type of relevant experimental information into a homogeneous structure that can be used directly for statistical correlation analysis by far outweighs the apparent absurdity of having executed a secondary protein structure prediction algorithm on sequences that *a priori *are never even transcribed into RNA, leave alone translated into protein. Furthermore, our information on transcribed sequences for instance is still incomplete – just consider the recent discoveries related to microRNAs – and hence a complete, unbiased probability annotation is more coherent [5]. Interestingly, a probabilistic framework also alleviates the problem of the formally undefined cause and effect relationship in the case of intrinsic stochasticity in the noisy experimental data by introducing the notion of fuzziness into the mapping; a process referred to as conditioning.

The nature of biological experimentation imposes two general constraints that need to be taken into account especially in the field of functional genomics. First, obviously, experimental information is never complete in that it is either a snap-shot of a dynamic reality, obtained as a mean measurement over large numbers of objects, biased by experimental or conceptual priors, or, most often, a combination of all the above, leading to context-dependency of the results. Second, the measurement itself introduces a non-negligible, albeit to some extent controllable, bias leading to further context-dependency of functional genomics data. Moreover, biological systems themselves display a strong context-dependency which is notably the object of study in functional genomics/systems biology: It is the combination of molecules in a cell that creates a biological function; hence the activity of a single molecule is context dependent. Thus, context-dependency of features is relevant for the comprehension of stimuli-responses and signals. Finally, context-dependency is itself question dependent. Consider the following example: Whether or not a given cell is differentiated to some defined state requires investigation of the presence of state-specific gene products and functionalities and the concomitant absence of molecules and functions specific to other cell-states. It does not, however, require any knowledge about the time dependency of the changes in gene expression and cellular physiology. A time series of experiments conducted on a differentiating cell, in this case, can therefore be simply projected, eliminating the time-dimension in addressing the question. The projection thereby has an important advantage over a simple end-point comparison, as (i) intermediate events are not omitted from the analysis, and (ii) statistical power is improved. However, when one tries to infer gene regulatory circuits, the time dimension of the experimental data is of outmost importance, whereas for instance the estimates of absolute molecular species quantities are far less important. Furthermore, the available genomic information can often be analyzed in a hierarchical manner. For certain biological questions it will not be important to have a detailed knowledge of feature probability profiles themselves but rather a more integrated, coarse-grained, combination of individual features. Ideally, by combining different features the set-theoretic conditioning can be turned into an unambiguous and well-defined cause and effect mapping. As studying different biological questions requires concomitant investigation of correlation and non-correlation, context-dependency and independency are similarly important. In conclusion, the very same set of information displays different context-dependencies as a function of the biological problem studied. We shall refer to this phenomenon from here on as "circumstantial context".

We develop here a mathematical approach to the quantification and statistical significance testing of context dependency in functional genomics data using our previously developed probability landscape framework. As context-dependency is not an absolute but a relative quantity, a flexible approach depending on the biological problem studied has to be realized. We furthermore demonstrate how according to the circumstantial context even very large numbers of individual landscapes stemming from experimental recordings can be merged into a single, collapsed profile with greatly improved statistical properties. This procedure can therefore be used in a systematic and controlled manner to reduce the computational and formal complexity of probability landscapes. Increased algorithmic efficiency and statistical power result jointly with heightened understanding of the biological mechanisms.

## Results

### Circumstantial probability profiles

Circumstantial context-dependency of functional genomics information does at the same time create important constraints, which need to be taken into consideration during statistical analysis, and simultaneously provides additional knowledge on the biological question studied. We have recently proposed probability landscapes as a means to integrate any relevant type of functional genomics information coherently and systematically into a structurally homogeneous object that can more easily be analyzed computationally. Here we asked whether or not the proposed structure of probability landscapes also permits systematic detection, analysis, and utilization of context-dependencies.

Let *X *be an observable quantity under investigation, taking either discrete, possibly symbolic, or continuous values. We have shown how experimental information on *X *can be expressed in a homogeneous and universal way as a genome-wide probability profile [5].

**Figure 1:**
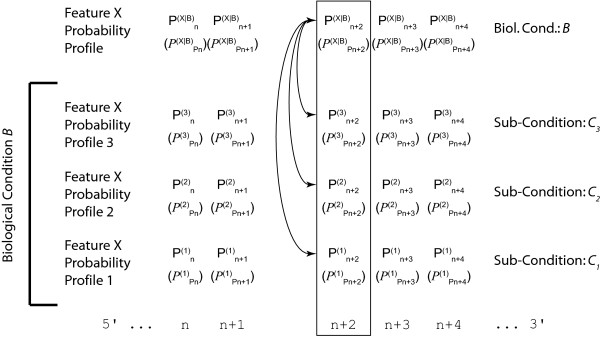
**Investigating context-dependency**. Point-wise comparison at a given genome location (the box underlines the location n+2) of probability profiles of a feature *X *obtained in condition *B *and under various additional prescriptions *C*_*i *_(*i *= 1, 2, 3) with the joint profile constructed from the pooled data. We have denoted in short $P_{n}^{\left(i\right)}=P_{n}^{\left(X|B,C_{i}\right)}$ and $P_{P_{n}}^{\left(i\right)}=P_{P_{n}}^{\left(X|B,C_{i}\right)}$ the 'probabilities of probability', i.e. the functional distributions describing the estimated variability of the distributions $P_{n}^{\left(i\right)}$. The comparison aims at determining whether the conditions *C*_*i *_provide additional information on *X *and decrease its indeterminacy or whether they can be ignored and the analysis performed on the pooled data. Essential conditions define the 'context' of the feature *X*.

**Figure 2:**
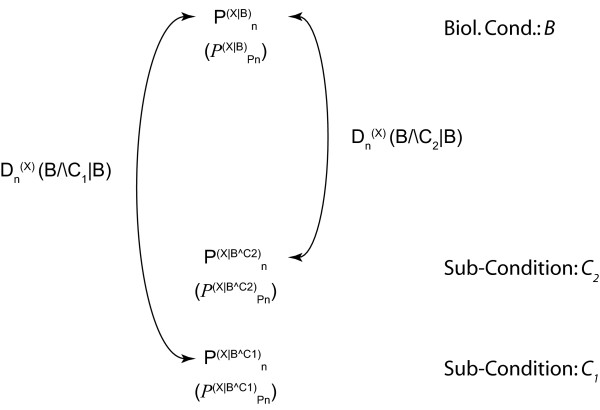
**Defining local distance measures between probability profiles**. For the validity of the methodology and an unambiguous interpretation of its results, it is essential to proceed hierarchically, and to compare distributions obtained from restricted groups of data, respectively in conditions *B*∧*C*_1 _and *B*∧*C*_2_, to the distribution obtained in the common biological condition *B *(pooled data). Each comparison is based on the computation of the Kullback-Leibler divergence $D_{n}^{\left(X\right)}(B\wedge C_{i}|B)$ between the distributions $P_{n}^{\left(X|B\wedge C_{i}\right)}$ and $P_{n}^{\left(X|B\right)}$. The significance of the comparison result depends on the variability of the distribution described by the functional distribution $P_{P_{n}}^{\left(X|B\wedge C_{i}\right)}$.

Given the biological nature of the information (see Background), probability profiles thus *de facto *involve conditional probabilities: *P*(*X*_*n *_= *x*|*B*) in case of a discrete-valued feature *X *or *ρ*(*X*_*n *_= *x*|*B*)*dx *in case of a continuous-valued feature *X*. We shall use $P_{n}^{\left(X|B\right)}$ to denote the probability at genome location *n *and *P*^(*X*|*B*) ^the corresponding probability profile over the genome (Figure 1). The conditions *B *correspond to the way of defining a subset of data, being more or less stringent on the similarity of the conditions (cell population, biological conditions) in which the data have been obtained. The conditions *B *could a priori include the subpopulation, various biological conditions, the timing along the cell cycle or the time lapse from the stimulus application. We actually adopt a hierarchical view: conditions *B *and sub-conditions *B*∧*C *constraining the conditioning *B *(Figure 2). Conditioning the landscape $P_{n}^{\left(X|B\wedge C\right)}$ with *B*∧*C *means that it has been constructed with a restricted set of data, i.e. a sub-group taken from the pool of data used to construct $P_{n}^{\left(X|B\right)}$ and satisfying the additional conditions *C*. It will appear essential for statistical inference to consider nested conditions *B *and *B*∧*C*. It is important to notice that the methodology we propose here is not intended to check whether conditions *C*_1 _and *C*_2 _are independent or not, but whether conditioning the feature *X *further by supplementing conditions *B *with additional constraints *C*, which effectively amounts to specifying a subgroup among the data recorded in conditions *B*, adds information on *X *and decreases its indeterminacy (Figure 2).

In all that follows, we shall consider a discrete-valued feature *X *for the sake of simplicity, without restricting the generality. Considering a continuous-valued feature requires only replacing ∑_x ∈ *χ *_by $\int_{x}dx$. Note that conditions considered here are those that can be controlled or selected at the experimental scale, i.e. at the cell population level. They are not precise enough to constrain each cell and its internal processes individually so as to determine *X *fully. In other words, whatever the prescribed conditions, the feature *X *remains a random variable and the mechanisms ruling its observed value still exhibit some stochasticity despite the conditioning; hence the probability distribution *P*^(*X*|*B*) ^remains non trivial. A description of how the construction of *P*^(*X*|*B*) ^can be achieved from feature probability profiles is found in the methods section and illustrated in Figure 1. The computation of *P*^(*X*|*B*) ^is achieved by combining the individual feature probabilities at any genome location n for different sub-conditions *C*_*i *_belonging to a biological condition *B*. This procedure can either be executed over defined intervals or the entire genomic sequence.

### Eliminating spurious conditioning, detecting essential ones

Considering the set of all the conditions that can be controlled or at least identified during the experiment, each feature will depend on some of these conditions whereas it will be independent of others (cf. Background). We thus want to determine for each biological question and each feature the subset of factors actually conditioning its probability landscape, and hence its effective context *C*(*X*). If *C*_*i *_does not add any information on *X*, it does not belong to the context *C*(*X*). Conversely, the proposed analysis allows features to be grouped in different subsets according to their circumstantial context.

Finding the effective, thus minimal, context *C*(*X*) among the full conditionings of *X *('minimax' entity) is a well-posed issue only in a hierarchical formulation: we have to investigate whether an additional condition *C *decreases the indeterminacy of *X *knowing *B*, and conversely whether data obtained under different conditions (*B*∧*C*_*j*_)_*j *_can be grouped into a single condition *B*∧*C *where *C *is the reunion of conditions (*C*_*j*_)_*j *_or even into the single condition *B *if (*C*_*j*_)_*j *_form a complete family, so that *C *adds in fact no additional prescription on *B*. This dual process can be iterated in both directions.

The issue is thus to compare *P*^(*X*|*B*) ^and *P*^(*X*|*B*∧*C*) ^to see whether the additional prescription *C *on the experimental conditions adds constraints and information on *X *(knowing *B*) or not (Figure 2). The issue has a very concrete and in practice essential outcome: providing a criterion to appreciate whether it is relevant to pool the data, or conversely whether some additional condition requires the set of data to be partitioned into sub-groups for a relevant analysis. Note that only explicitly controlled or described conditions, of which the experimentalist is aware, can be mentioned in the probabilities. A wealth of implicit conditions is also present, and one of the aims of this work is to develop a coherent way to bring forward the relevant ones. In confronting two probability landscapes *P*^(*X*|*B*,1) ^and *P*^(*X*|*B*,2) ^constructed from data recorded independently, one might guess that an additional condition *C *is present, that explains the discrepancy between the two landscapes, if any: $P^{\left(X|B,i\right)}=P^{\left(X|B,C_{i}\right)}$.

### Divergence of probability profiles

At each genome location *n*, the probabilities $P_{n}^{\left(X|B\right)}$ and $P_{n}^{\left(X|B\wedge C\right)}$ are defined on the same space (the state space *χ *of the feature *X*). Various ways of measuring the discrepancy between these probability distributions can be considered: distance sup on *χ*, distances associated with the *L*_*p *_norm, or distance in the parameter space if the distributions can be parameterized. We rather choose minus the relative entropy, known as the *Kullback-Leibler divergence *(it is indeed not strictly a distance because of its asymmetry) [6]. A detailed description of the calculation is found in the Methods section, where we define the divergence measure $D_{n}^{\left(X\right)}(B\wedge C|B)$ between $P_{n}^{\left(X|B\wedge C\right)}$ and $P_{n}^{\left(X|B\right)}$ (Methods, Figure 2).

Note that it is meaningless to compare $P_{n}^{\left(X|C_{1}\right)}$ and $P_{n}^{\left(X|C_{2}\right)}$ where *C*_1 _and *C*_2 _are disjoint conditions. Indeed, it would be impossible to disentangle the relative contributions of *C*_1 _and *C*_2 _and the actual origin of a difference (or a similarity) between $P_{n}^{\left(X|C_{1}\right)}$ and $P_{n}^{\left(X|C_{2}\right)}$. Our analysis relies on the hierarchical structure of conditions and sub-conditions, of which the (ir)relevance is investigated.

**Figure 3:**
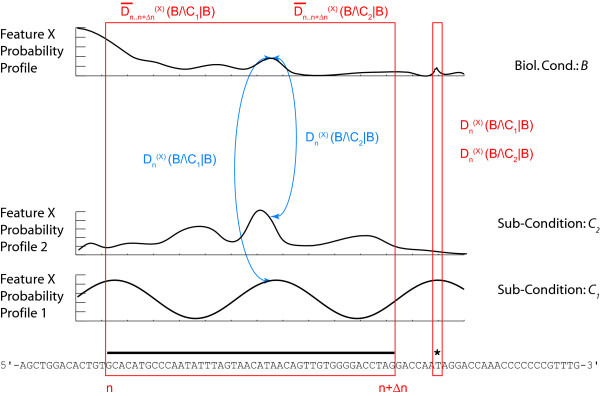
**Local and extended divergence**. From the knowledge of the point-wise distances $D_{n}^{\left(X\right)}(B\wedge C_{i}|B)$ (right box) an integrated comparison of the landscapes is performed by computing either and average distance or a cumulative distance ${\bar{D}}_{\left[n,n+\Delta n\right]}^{\left(X\right)}(B\wedge C_{i}|B)$ (left box) as a weighted sum of the distances ${\left[D_{j}^{\left(X\right)}\right]}_{n\leq j\leq j+\Delta n}$. This procedure allows the extended sequence features (such as an exon for instance, black bar above nucleotide sequence) to be treated in a coherent manner. Individual nucleotide features (such as SNP data for instance), are compared directly (right box).

In the case that the feature probability profiles $P_{n}^{\left(X|B\wedge C_{i}\right)}$ for the sub-conditions *C*_*i *_have been recorded with no memory of the original data, the reference landscape $P_{n}^{\left(X|B\right)}$ cannot be obtained by directly pooling the data, but should be first computed by pooling the profiles $P_{n}^{\left(X|B\wedge C_{i}\right)}$ using a weighted average, with weights proportional to the rarity of conditions *C*_*i*_. Then each probability profile $P_{n}^{\left(X|B\wedge C_{i}\right)}$ can be compared to $P_{n}^{\left(X|B\right)}$ in order to assess whether the sub-condition *C*_*i *_adds significant information or not (Figures 2, 3). Please note that the figures are just a schematic illustration and do not correspond to concrete values. We give an example of Kullback-Leibler divergence on real transcriptome data at the end of the Results section. The black (Figure 2) and blue (Figure 3) arrows indicate the divergence at a given position *n *between the two feature probability profiles and the collapsed profile. This divergence can either be exploited locally at any position *n *(as illustrated in Figure 2, and by the narrow red box to the right of Figure 3), or over an entire interval of genomic sequence (large red box, interval *n*..*n*+Δ*n*, Figure 3).

### Statistical significance testing

The Kullback-Leibler divergence thus provides a tool for calculating the difference of the individual conditional feature probability profiles $P_{n}^{\left(X|B\wedge C_{i}\right)}$ with the coarser-conditioned probability profile $P_{n}^{\left(X|B\right)}$. The divergence is neither upper-bound, nor has any absolute bearing. The question of how to judge a Kullback-Leibler divergence of sufficient magnitude in order to decide or not to collapse different feature probability profiles is hence not trivial (Figure 3). Either a set of arbitrary thresholds has to be defined, possibly by working with large numbers of actual datasets from well defined biological conditions, or a statistical test has to be developed. Obviously, the latter should be given strong preference. In order to do so, one has to compute probabilities of neighborhoods of the distributions $P_{n}^{\left(X|B\right)}$ and $P_{n}^{\left(X|B\wedge C\right)}$ using the previously defined 'probabilities of probabilities' (functional distributions, Lesne & Benecke 2008) $P_{P_{n}}$. A possible way would be to compute

$$\begin{array}{ccc} P_{P_{n}}^{\left(X|B\wedge C\right)}\left[V\left(P_{n}^{\left(X|B\right)},\varepsilon \right)\right] & \text{and} & P_{P_{n}}^{\left(X|B\right)}\left[V\left(P_{n}^{\left(X|B\wedge C\right)},\varepsilon \right)\right] \end{array}$$

where *V*(*P*_*n*_, *ε*) is the ball of radius centered on the distribution *P*_*n *_(distribution over the space *χ*); it is thus a neighborhood in a functional space, where the radius bounds the Kullback-Leibler divergence between an element and the center of the ball. We have recently investigated for a more general case how conjoint statistical significance testing for similarity and distinctness can be achieved on such a measure. Please refer for a more detailed description of the methodology to [7]. Briefly, any experimentally obtained signal (such as the fluorescence/chemiluminescence signal of a spot on a microarray) is interpreted as a random independent sample of some random variable, assumed normally distributed and with unknown average. The mean and variance estimates can be used to construct an unbiased maximum likelihood estimator, which is itself a random variable of Gaussian form. In order to formulate quantitative statements concerning the relative differences between different biological conditions, we introduce a cone *C*_*α *_over the first diagonal of a signal estimate under two different biological conditions with half-angle *α*. The rationale for considering such cones rather than homogeneous error margins is to control the relative error. Using the so-called ratio distribution for independent normal distributions, we can then determine a likelihood of the mean estimates being within a distance smaller than *C*_*α *_or not of the actual mean of the random variable. This distance measure is symmetric in the sense that we can estimate both similarity and distinctness. Moreover, the measure is also amendable to testing for statistical significance using serialized two-sided T-tests. By defining a single confidence interval on the above measure the decision on whether or not to collapse feature probability profiles then becomes straight-forward. Interestingly, the significance testing of distinctness and similarity, as we develop it in [7], takes into account the relative variance over the measure in case of massive-parallel data such as functional genomics experimental observations in form of the half-angle *α *of the cone *C*_*α*_. In this case the quality, or better statistically perceived quality, of the measure on the observable under different biological conditions is directly taken into consideration when estimating the statistical significance of the Kullback-Leibler divergence.

### Extending the divergence analysis over the genome

**Figure 4:**
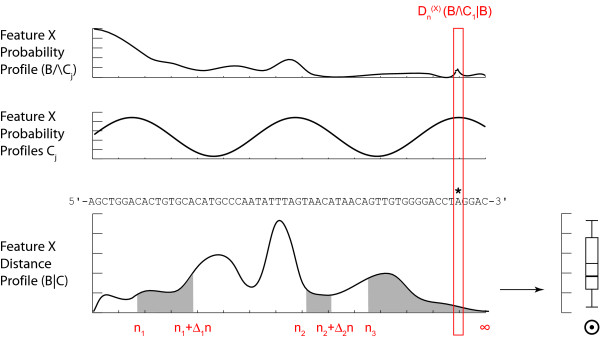
**Integration of distance profiles**. The local distance measure $D_{n}^{\left(X\right)}(B\wedge C_{i}|B)$ is computed over the entire profile length (genome). Unlike the individual feature probability profiles, the distance profile can be integrated to give rise to a meaningful genome wide distance measure. The proper integrated distance ${\bar{D}}_{I}^{\left(X\right)}$ might involve several genome intervals *I *= [*n*_1_, *n*_1 _+ Δ*n*_1_] ∪ [*n*_2_, *n*_2 _+ Δ*n*_2_] and/or an "infinite" interval [*n*_3_, + ∞[. Obviously, other genome wide measures can be defined for the divergence such as the mean, median, sup, min, etc. Again, the divergence measure need not to be computed over all nucleotides but might be restricted to any combination of non-overlapping intervals *I *or individual positions *n*. In this way the global divergence measure computation can be restricted to particular sequence features such as coding regions.

So far we have only discussed the context-dependency analysis locally; that is at any genome position *n*. As feature probability profiles extend over the entire genomic sequence of the organism under study, a generalization is required, which as shown below is straight-forward in our approach. Consider the case where a subset of feature probability profiles is known on biological grounds to reflect relevant measures on the biological and physical properties of a stretch *I *of the genome (e.g. the linear extension of a gene, possibly with gaps, such as transcriptome data, Figure 3). We compute for each *n *∈ *I *a distance $D_{n}^{\left(X\right)}(B\wedge C|B)$ between the distributions conditioned respectively by *B *and *B*∧*C*. Then for instance the average distance ${\bar{D}}_{I}^{\left(X\right)}(B\wedge C|B)$ or cumulative distance $A_{I}^{\left(X\right)}(B\wedge C|B)$ can be easily defined (Figure 3). Other possibilities exist such as the sup. Averaging over the genome locations *n *over a window Δ*n *of relevance for *X *(*X*-dependent window size), yields an average distance ${\bar{D}}_{\left[n,n+\Delta n\right]}^{\left(X\right)}(B\wedge C|B)$. Depending on the nature of the features, and exploiting the fact that unlike the feature probability profiles distance profiles can be directly integrated, a more meaningful index is to integrate the distance $D_{n}^{\left(X\right)}(B\wedge C|B)$ over the relevant window, yielding the integrated distance $A_{\left[n,n+\Delta n\right]}^{\left(X\right)}(B\wedge C|B)$. Averaging or integrating over relevant windows *I *can be achieved locally or globally over the entire chromosome or genome (Figure 4). Importantly, and again depending on the biological question posed, the divergence calculation can also be performed serially or cumulatively over different *I*_*j *_intervals. Finally, the measures over the different intervals *I*_*j *_can be weighted as well if reasonable (Figure 4). Different measures for the integrated Kullback-Leibler divergence can also be defined such as the maximum, minimum, mean, median, quantile, or combinations thereof, whether weighted or not. The box-plot in Figure 4 serves simply to illustrate this fact. Additional measures can certainly be found. Their significance will have to be defined according to the biological problem under study, the nature of the experimental data, and the underlying reasoning for the Kullback-Leibler divergence approach in the concrete example under scrutiny. In the example we develop on real transcriptome data (see below), we use the median.

### Circumstantial and hierarchical complexity reduction

**Figure 5:**
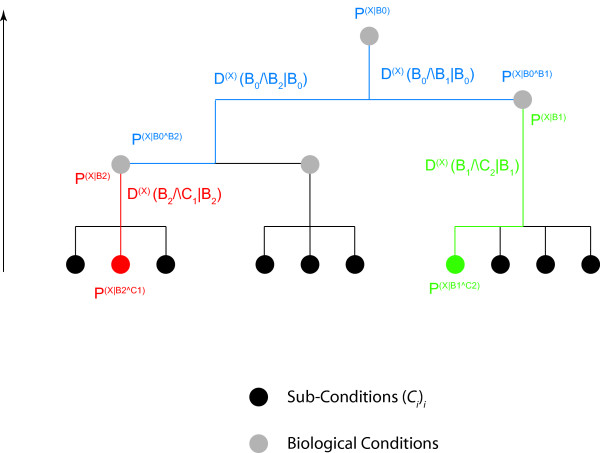
**Feature probability quality profile construction for experimental data**. The set of conditions that are essential for feature *X *are determined hierarchically, either by considering more detailed prescriptions (additional disjoint conditions (*C*_*i*_)_*i*_) corresponding to a partition of the data in constructing the conditional profiles, or in aggregating the conditions if the conditions (*C*_*i*_)_*i *_have no impact on the feature. This procedure can be performed recursively. Once sub-conditions have been collapsed to a biological condition, the biological condition can be compared using the same logic to the next higher level biological condition. Please note that for reasons of simplicity we only consider the two immediately concerned levels explicitly in the notation. Imagine for instance data pertaining to the transcriptome of different types of blood cells (*C*_*i*_)_*i*_. One might want to consider every cell type individually, or the red and white blood cells (*B*_1_, *B*_2_) jointly or the entire compartment (*B*_0_).

**Figure 6:**
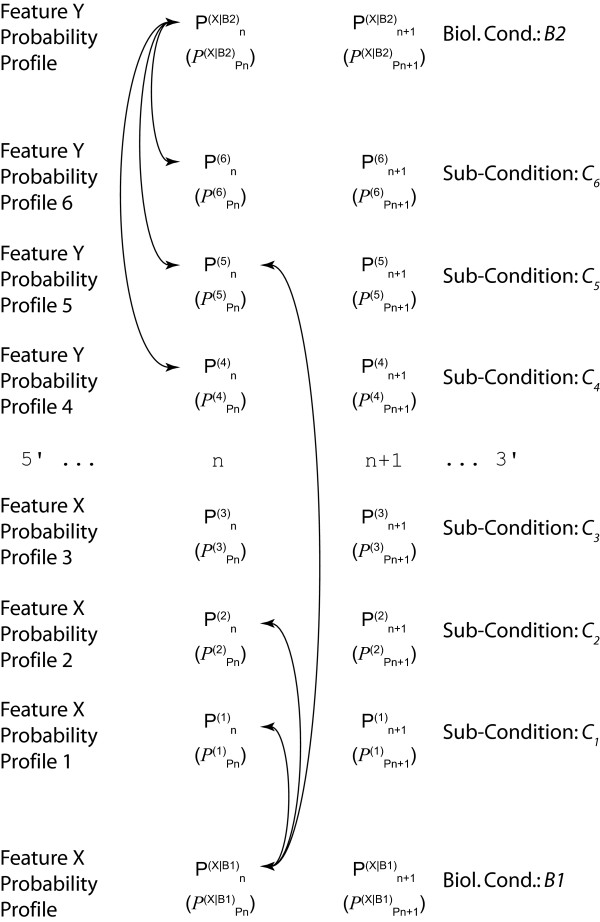
**Flexible, question-driven profile collapse**. The context-dependency analysis is question dependent, and hence needs to be performed for each question individually. Thereby, individual sub-conditions can be combined in a non-exclusive manner as a function of their circumstantial context.

As discussed throughout this work, context-dependency of features is itself dependent on the biological question addressed. Given a biological question or context, any set of context-dependent conditions can be tested against a cumulative biological condition calculated as an average measure over the set of sub-conditions for its relative contribution to the overall information. This can be achieved in parallel for as many different (sub-)conditions as available. The relevance of any feature probability profile with respect to the biological question addressed is hereby and importantly solely defined through a statistical significance measure in the information theoretical divergence from the pooled information when considering larger and larger joint sets of conditions. This procedure can be hierarchically repeated (using a single confidence interval) to conditionally collapse individual profiles further and further (Figure 5). The schematic representation of different conditioned feature probability profiles, their inter-relationship, and the natural hierarchy of the different probability profiles with respect to a biological condition *B *are illustrated. Wherever the statistical significance of the distance measure exceeds a defined threshold the distance is considered insufficient to warrant the sub-condition being analyzed separately, and thus the corresponding profiles are collapsed. This procedure can be performed recursively. Consider for example the question of what the transcribed sequences in a given genome are (notably without any restriction of a particular biological condition). If one uses the many thousands of available microarray transcriptome studies, or in the near future, high throughput sequencing transcriptome data, which were all recorded under precise experimental and thus biological conditions, no significant context-dependency arises through the choice of the appropriate biological conditioning. Thus, all existing transcriptome data would successively be collapsed to give a single feature probability profile that could directly be seen as a probability of any nucleotide in the genome being transcribed (obviously only provided sufficiently divergent transcriptome data are available). Such an optimally conditioned profile could subsequently be used to search for correlations between the genomic sequence and the occurrences of all expressed sequences in order to search for sequence elements statistically significantly associated with transcribed sequences. While this example, as extreme as it is, might not seem appropriate, just consider that any level of acceptable divergence can be defined with respect to the biological question addressed, and that feature probability profiles can be regrouped into any number of not necessarily exclusive subsets the experimentator sees fit (Figure 6). Therefore, a continuum of nested profiles ranking from individual feature profiles to a totally collapsed landscape exists. This continuum needs to be explored for every biological question separately, which is why the complexity of the landscape can not be reduced permanently. Essentially, for every new investigation of the structure, the feature probability landscape is at first totally uncompressed, and using the method described here, is then locally – with respect to the sub-conditions *C*_*i *_– collapsed as a function of the biological conditions *B*_*j*_. Different biological conditions *B *will lead to different combinations of *C*_*i *_profiles being collapsed (Figure 6). Genome probability landscapes are therefore a dynamic structure that can be locally collapsed as a function of the circumstantial context.

### Circumstantial context illustrated with a theoretical example

**Figure 7:**
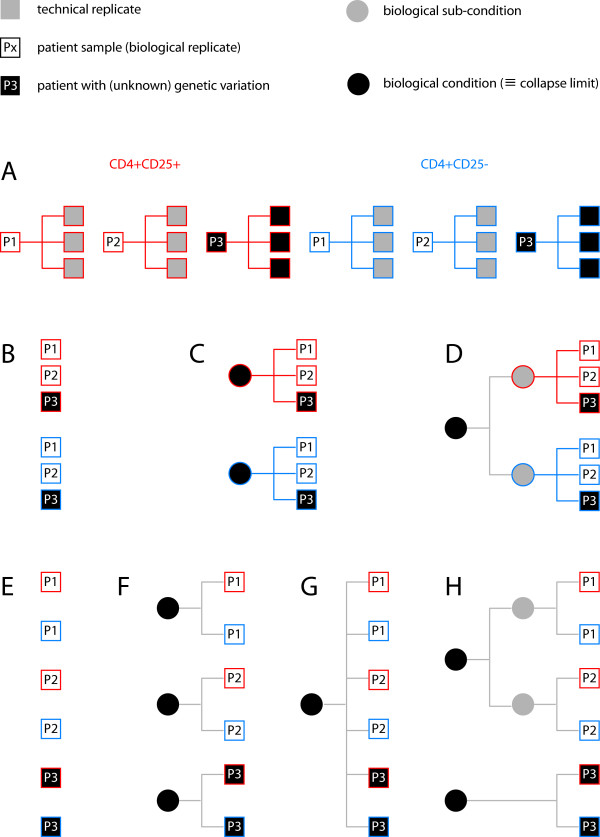
**A theoretical example of circumstantial context**. (A) Let *Px *be a subject from whom a blood sample has been drawn. CD4+CD25+, CD4+CD25- indicate the T-cell subpopulations for which transcriptome profiles have been recorded. Subject *P3 *carries an unknown genetic variant with limited but functional implication for the expression of some genes. The technical variability of the experiments is sufficiently small to warrant calculation of mean expression profiles. Depending on the circumstantial context either inter-cell type comparisons can be performed in a context-dependent manner (B-D) or subject heterogeneities can be studied (E-H). In either case the divergence between features, and therefore the context-dependency, will determine to what degree the probability profiles can be collapsed upon one another. (Please refer to the discussion section for a detailed description).

In order to illustrate the applicability of the methodology developed here let us consider the theoretical example of an analysis of different T-cell populations from a plausible human patient study for how context-dependency analysis is performed in a biological question motivated manner (Figure 7).

Let *Px *(*x *= 1, 2, 3) be a subject from whom a blood sample has been drawn. The peripheral blood mononuclear cell (PBMC) population has subsequently been separated by fluorescence activated cell sorting (FACS) and the two T-cell subpopulations CD4+CD25+, CD4+CD25- were enriched using the corresponding cell surface markers. Assume furthermore that the CD4+CD25+ (red) and CD4+CD25- (blue) cells, which are both involved for instance in the inflammatory response, have undergone brief exposure to an inflammation inducing agent such as an interleukin during *ex vivo *primary cell culture, before the cells were harvested and total RNA was extracted for transcriptome analysis using several technical replicates per subject (Figure 7A). Finally, assume that subject *P3 *carries an unknown genetic variant with limited but functional implication for the expression of some genes. For simplicity, consider the technical variability of the experiment to be sufficiently small to warrant the calculation of mean expression profiles for each T-cell subtype from each subject.

Several biological questions might be addressed using such a dataset. The first set of questions could relate to the difference in the transcriptional responses of CD4+CD25+ and CD4+CD25- T-cells to stimulation using the interleukin (Figure 7B–D). Depending on the statistical significance of the Kullback-Leibler divergence between the different transcriptome probability profiles of the subjects in either the CD4+CD25+ or the CD4+CD25- cases (and therefore the heterogeneity between individuals), the probability profiles might either need to be considered separately (Figure 7B) or can be collapsed to a CD4+CD25+ and CD4+CD25- probability profile (Figure 7C). Note that any other combination of the data into subsets is theoretically possible as well. In the latter case (Figure 7C) one would conclude that the biological variability between subjects is sufficiently small with respect to the difference of the two cell-types to be neglected. Now assume that you restrict your analysis to genes targeted by the interferon gamma (IFN*γ*) pathway which we shall consider equally active in both T-cell populations. In this case the Kullback-Leibler divergence calculated exclusively over the IFN*γ *target gene subset might indicate that indeed the probability profiles of all six samples (across subjects and across cell types) might be collapsed to give rise to a single profile (Figure 7D). This total collapse of the data however and importantly has been only calculated on, and therefore is only valid for, the IFN*γ *regulated subset of genes. These two examples justify the fact that feature probability profile complexity reduction is dependent of the biological phenomenon under study and the specific context. The example can be extended to the analysis of inter-subject variation (Figure 7E–H) independent of T-cell subpopulation. Again, the Kullback-Leibler divergence analysis will provide a statistically sound argument to either analyze the probability profiles individually (Figure 7E), collapse the two probability profiles available for each subject (Figure 7F), or combine all profiles into one (Figure 7G), or any combination thereof. Note that although the result of the operation shown in Figure 7D and 7G might appear to be identical, this is not the case as the statistical analysis leading to these similar results is based on distinct quantities: in the former case the similarity between gene expression responses between different cell types; in the latter the similarity between different individuals. Finally, assume that the genetic variation in subject *P3 *affects IFN*γ *signaling (which could be the case in some auto-immune disorders like allergy). It is reasonable to believe that if you were to restrict your analysis to the IFN*γ *pathway as above (Figure 7D) you might find the analysis based on the Kullback-Leibler divergence to exceed the statistical significance threshold and hence to warrant separate analysis of the regrouped profiles from subjects *P1 *and *P2 *versus subject *P3 *(Figure 7H). Again, context-dependency and circumstantial context will require different analysis strategies.

### Circumstantial context analysis on actual transcriptome data

**Figure 8:**
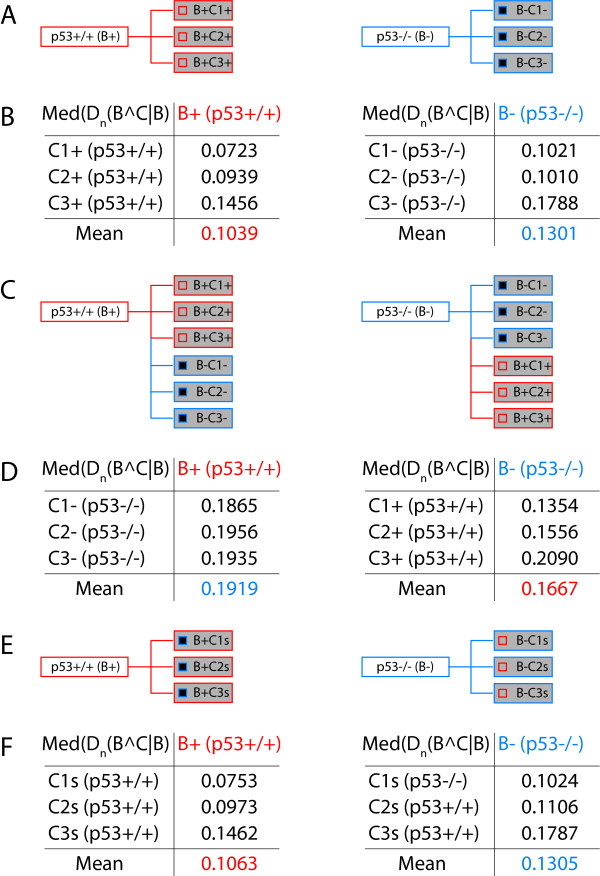
**A concrete example of circumstantial context analysis using transcriptome data**. (A) Schematic representation of the two biological conditions (B+ p53+/+, B- p53-/-), and the three biological replicates (C1, C2, C3) from the published study [8]. The small squares inside the rectangles for the biological replicates represent the 899 probes that are statistically significantly regulated between the two biological conditions and should be considered p53 regulated genes. (B) Median of the Kullback-Leibler divergence measures for the indicated comparisons. The mean of the median of the divergence for the three comparisons is also indicated. (C) Schematic illustration of the subsequent divergence analysis, where the biological replicates of one biological condition are analyzed with respect to the other biological condition. (D) The data for the experiment illustrated in (C) are shown in a similar manner to (B). (E) The probability profiles for the 899 p53 statistically significantly regulated probes were swapped between the two biological conditions. (Compare the small squares inside the rectangles of the biological replicates). (F) Results for the experiment illustrated in (E) as in (B, D). The data should be compared to the data in (B).

**Figure 9:**
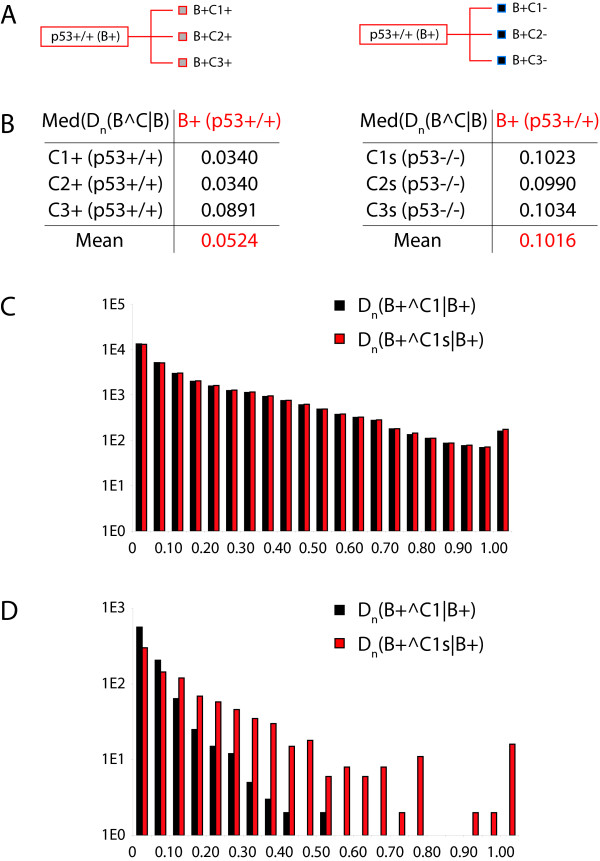
**Selective versus global circumstantial context analysis at the example of actual data**. (A) The Kullback-Leibler divergence measures were once calculated for the 899 p53 sensitive probes in the B+ case, and once using the B- probability profiles compared to the B+ biological condition. (B) Data for (A) as in (Figure 8B, D, F). Both tables can be directly compared. (C) Histogram of the Kullback-Leibler divergence distribution over the entire set of 31710 probes analyzed in the two indicated cases. Note that "C1mix" refers to the swapping experiment as illustrated in (Figure 8E). Note also that the final bin encompasses the interval]1..+∞[. (D) Similar histogram as in (C) for the 899 probes showing significant p53 regulation (compare (A)).

To demonstrate practical applicability of our approach we present here an analysis of circumstantial context at a concrete example of transcriptome data. The dataset we used was recently generated in our laboratory and has been published [8]. All microarray experiments discussed hereafter are available from the GEO database using accession number GSE10795 (see also Methods). In [8] we present a transcriptome analysis of the apoptotic transcription program downstream of the delta splice-isoform of the TFIID associated factor TAF6*δ *in two human isogenic cell lines inactivated or not for the p53 gene. Briefly, we demonstrate that TAF6*δ *acts downstream and independently of p53 to control gene expression at the onset of apoptosis [8]. For the following demonstration we selected six experiments: GSM272658-60 (TAF6*δ *induction in the p53-/- background, hereafter referred to as biological condition B-, using three independent biological replicates referred to as C1-, C2-, and C3-), and GSM272664-6 (TAF6*δ *induction in the p53+/+ background, hereafter referred to as biological condition B+, using three independent biological replicates referred to as C1+, C2+, and C3+). The data were processed as described in the Methods section and in [5] in order to obtain probability profiles, and subsequently we calculated the Kullback-Leibler divergence at probe resolution for different contexts (Figures 8 &9). Note that certain simplifications were introduced into the calculation of the probability profiles. Those modifications are described and justified in the Methods section, and reflect the limited scope of the analysis presented here (focusing on the circumstantial context only), and the very limited amount of data used, sufficient for the demonstration but very far from fully exploiting the wider concept of probability landscapes. The corresponding data for the analysis discussed below are to be found as additional files SupDataFile01.txt, SupDataFile02.txt, and SupDataFile03.txt.

As shown in Figure 8A and 8B, we have first calculated the Kullback-Leibler divergence for the individual biological replicates versus a collapsed probability profile for the entire biological condition. As very few datasets were used, neither the calculation of statistical significance of individual divergences between the different biological replicates and the collapsed probability, nor the statistical significance of differences between the Kullback-Leibler divergence distributions was exploited, and we simply use the median of the divergences as well as its mean over a set of comparisons as comparative measures (Figure 8B). Having compared the individual biological replicates to the corresponding integrated probability profile of the biological condition, we also investigated the respective divergence distributions obtained when comparing the Ci+ of B+ to B- and vice versa the Ci- of B- to B+ (Figure 8C &8D). As can be easily appreciated, in all cases the divergence increases as would be expected for data from different biological conditions. The increase in the means for instance might appear relatively modest, but given the distribution of the Kullback-Leibler divergences (see for instance the histogram in Figure 9C), such differences are probably indeed significant. As mentioned above, a statistical analysis would, however, require a much larger dataset. We then decided to do two experiments in order to substantiate the claims made above using the theoretical example (Figure 7). First, we swapped the probabilities associated with 899 probes that we had previously found to detect statistically significant changes in gene expression when comparing the B+ (p53+/+) and B- (p53-/-) biological conditions [8]. In order to do so the probabilities calculated for the corresponding probes from Ci+ were assigned to the same probe in Ci- and vice versa (Figure 8E, indicated by the addition of "s" to the biological condition identifier). We thus exchanged 2.8% of the entire probability profile with its counterpart from the other biological condition. The corresponding divergence measures are shown in Figure 8F. As can be seen by comparison with the values in Figure 8B, we observe a modest increase of the Kullback-Leibler divergences, which, however, should – at least given the sample size – not be considered significant. Therefore, and unlike swapping the entire profiles (Figure 8C &8D), such a restricted modification of the profiles is not necessarily detectable (compare also our discussion of Figure 7G in the preceding section). If, however, one restricts the analysis of the Kullback-Leibler divergence to the 899 probes only (*cf. *our discussion of extending the analysis over the genome, Figures 3 &4), measurable differences between the normal and swapped situations again occur (Figure 9A &9B). These differences are the more striking if one compares the histograms over the entire divergence distribution for the first experiment (Figure 8E) and the second (Figure 9A) with their non-modified counterparts, as shown in Figure 9C and 9D. Whereas, in the first case where the 899 swapped probabilities have an almost undetectable effect on the median as well as the entire distribution (Figure 9D), the case where only the 899 probes are considered in isolation not only shows an increase in the median, but also a starkly modified overall distribution (Figure 9C). Note that both histograms are on a log scale and that the last bin encompasses all values greater than unity. Therefore, and as we had pointed out in our theoretical discussion of the properties of the Kullback-Leibler divergence and circumstantial context above, the biological question will condition the decision whether or not to collapse several profiles into one. Concretely, if one were exclusively interested in studying the p53 responsive genes in above dataset, as the latter swapping case demonstrates, a complexity reduction would not be advisable, whereas on the other hand, when studying the entire genomic response to the stimulus, the divergence over the swapped p53 target gene responses would not significantly affect the outcome of the analysis. This illustrates the applicability and feasibility of the methodology we develop here.

## Discussion

We have introduced probability landscapes as a homogeneous and formally consistent representation of any type of functional genomics information in order to achieve a unique structure that can statistically be systematically interrogated using correlation measures [5]. To reduce unnecessary formal, mathematical and computational complexity we propose here to use the existing *de facto *context-dependency of features as a question-dependent measure for collapsing subsets of the landscapes. Consider the case where *C*_*i *_refer to sub-conditions of the circumstantial context of the biological condition *B *in which the feature *X *has been recorded (Figure 1). We want to know whether it is necessary to consider them as distinct populations or whether it is meaningful to pool them. We pool the local measures $P_{n}^{\left(X|B\wedge C_{1}\right)}$ to $P_{n}^{\left(X|B\wedge C_{j}\right)}$ into a combined measure $P_{n}^{\left(X|B\right)}$ (Methods) using a weighted average accounting for the presumed frequency of these sub-populations and possibly of the quality (weighting by the inverse standard deviation) of the measurements (Figure 2). This is repeated over the entire genome sequence to give a global profile *P*(^*X*|*B*) ^using the Kullback-Leibler divergence (Figure 3) and either any – possibly weighted – combination of subsequences or the entire genome (red boxes in Figure 3). Thereby, not only can the subsequences over which the divergence is determined be freely chosen, but also the feature divergence profile of a biological condition *B *can be analyzed in a continuous way over the entire genome or defined intervals (such as gene sequences) by integrating over the corresponding (sub-)sequences (Figure 4). This genome-wide distance measure is meaningful, unlike the individual feature profiles. Using a statistical significance test, for any individual feature probability profile $P^{\left(X|B\wedge C_{i}\right)}$ the relative contribution of *C*_*i *_to conditioning can be calculated. If the conditioning by any *C*_*i *_leads to a statistically significant divergence (suggesting that the associated sub-population is well delineated and has a specific signature as regards the feature *X*) the profile is kept as separate entity. In contrast, if statistical significance is not reached, the condition *C*_*i *_is considered inappropriate with respect to the biological question posed as it does not provide a measurable constraint on the value of the feature *X *and can be combined with any other statistically insignificant *C*_*j *≠ *i *_into a biological condition *B *feature probability profile, thereby collapsing part of the landscape (Methods). Two advantages arise in this case: (i) the complexity of the structure is reduced in a controlled and with respect to the biological question asked irrelevant manner, and (ii) the statistical power of the feature probability profile *P*^(*X*|*B*) ^is increased with respect to individual $P^{\left(X|B\wedge C_{i}\right)}$. This procedure can be performed at any interesting scale or functional level as feature probability profiles can be organized into a hierarchical structure with respect to the biological question, and thus the probability landscape over the genomic sequence can be reduced in complexity until all remaining context-dependencies reach statistical significance at which an optimum for computational complexity and statistical power is reached (Figure 5). Different biological conditions can thereby be defined with maximum flexibility using separate or overlapping subsets of sub-conditions (Figure 6).

Note that since we are comparing the distributions of the same random variable under different conditions, it is only the distance (or divergence) between the two distributions that is meaningful. A joint probability, such as mutual information, can not be envisioned. This also holds for the case of two different variables because the joint probability distribution is inaccessible. Eventually, one could envision considering mutual information in the context of the comparison of two probability distributions (rather than individual variables), thereby rejoining the concept of probabilities of probabilities we have previously developed [5]. However, this seems impractical in concrete terms.

The methodology developed here represents a systematic and simple way of testing the statistical limits of complexity-reduction and hence explanatory power of the integrative genomics data in their respective contexts (see for instance Figures 7 &8). We note that our method represents an application of concepts related to context-trees to the probability landscape idea. Circumstantial context analysis and landscape collapse thereby operate in similar manners to Markov chains with variable length for the analysis of time-series from t_0 _to -∞ (which can be considered the historic context) [9]. Markov chains and Hidden Markov Models (HMMs) have found wide-spread application in the analysis of genomic and gene sequences ([10] and references therein). In contrast to our approach, however, the probabilities assigned to individual nucleotides here reflect the linear sequence context ("horizontal" analysis of sequence statistics) whereas the probability landscape concept we advocate uses the nucleotide based probabilities to integrate "vertically" sequence-dependent features such as activity. Both approaches share common ideas such as the use of probabilities and a single nucleotide resolution, but they differ significantly in their scope and methodology. HMMs are for instance quite constrained in that they require sequentiality (making them particularly interesting in the studies of sequences) and restricted in the number of sequential objects/variables under study. It does not at all seem feasible to develop HMM approaches for entire genomes. Probability landscapes, in contrast, neither require sequential organization *per se*, nor are limited in the number of objects under study as they can be decomposed. The complexity-reduction procedure for probability landscapes developed here can also be seen as an illustration of both of these features. It is therefore quite obvious that HMMs and probability landscapes are independent though complementary concepts that should acquire synergistic roles in genome analysis.

We also note that the Kullback-Leibler divergence calculation provides measures that can be used directly for clustering of probability profiles. Clustering of probability profiles might help to establish and analyze relatedness among data otherwise not compared directly.

## Conclusion

Feature context-dependency can be systematically investigated using probability landscapes. Furthermore, different, independent feature probability profiles can be collapsed as a function of circumstantial context to reduce the computational and formal complexity of probability landscapes. Interestingly, the possibility of complexity reduction can be linked directly to the analysis of context-dependency. Furthermore, as the criteria for circumstantial complexity reduction are statistically controlled, an optimal probability landscape is created in a biological question dependent manner. These two advances in our understanding of the properties of probability landscapes not only simplify subsequent cross-correlation analysis in hypothesis-driven model building and testing, but also provide additional insights into the biological gene regulatory problems studied. The nature of individual features can be probed with respect to posed problems and a classification of features according to their respective contexts can be achieved. Therefore, increased algorithmic efficiency and statistical power result jointly with heightened understanding of the biological mechanisms. Obviously, other features of circumstantial context and probability landscapes in general still remain to be fully exploited.

## Methods

### Constructing $P_{P_{n}}$

In cases where the feature *X *takes discrete values, the construction of $P_{P_{n}}^{\left(X\right)}$ has been detailed in [5]. For continuously valued *X*, hence where the probability $P_{n}^{\left(X\right)}$ is defined as a density, the probability of probability $P_{P_{n}}^{\left(X\right)}$ is a functional probability distribution, the construction of which will basically follow the same steps as for the Wiener measure and defines a mathematical object of the same nature.

Another option is to discretize the feature *X*, using e.g. thresholds or any biologically meaningful partition of the range of values of *X *so that $P_{n}^{\left(X\right)}$ becomes a finite array of probabilities (summing up to 1) and the distribution of the probability distribution $P_{P_{n}}^{\left(X\right)}$ describes the experimental and statistical variabilities on the estimate of this array.

Still another option to construct $P_{P_{n}}^{\left(X\right)}$ is to discretize the probability profile itself, using e.g. statistically meaningful threshold to partition the range of values of the density and replace $P_{n}^{\left(X\right)}$ at each nucleotide position *n *by an array of numbers. At this point, it might be more relevant to consider the cumulative distribution (i.e. defining quantiles) and the probability of probability now describes the distribution of errors on these quantiles.

Note that discretization procedures involve extra knowledge that is at the same time a flaw (introducing some subjectivity if not arbitrariness in the description and analysis) and an advantage (it reduces a wealth of information in an intractable high-dimensional space to a finite number of clear-cut and discrete, e.g. binary, properties, and takes benefit of all the additional knowledge available, e.g. on biological grounds, on the system). To enhance the beneficial aspect while minimizing the drawback, it is then essential to perform a discretization for each specific question and setting, extracting the minimal information that is relevant for that question.

### Collapse of conditional profiles

When the comparison of the profile $P^{\left(X|B\wedge C_{i}\right)}$ with *P*^(*X*|*B*) ^shows that the condition *C*_*i *_has no statistically significant impact as measured using the Kullback-Leibler divergence (see below) on the feature *X *(at least in conditioning *B*), for each element of a set of complementary conditions (*C*_*i*_)_*i*_, the complexity of the probability landscape can be reduced by collapsing the profiles $P^{\left(X|B\wedge C_{i}\right)}$ into *P*^(*X*|*B*) ^by computing a weighted average $\sum_{i}\omega_{i}P^{\left(X|B\wedge C_{i}\right)}$. The weights *ω*_*i *_(with $\sum_{i}\omega_{i}=1$) are chosen either according to the sizes of the data pools in conditions *B*∧*C*_*i *_respectively, or to the variability of the profile (*ω*_*i *_being inverse proportional to the standard deviation). The procedure can be executed recursively.

### Proof of the absolute continuity of *P*^(*X*|*B*∧*C*) ^with respect to *P*^(*X*|*B*)^

In cases where the feature *X *takes discrete values

*P*^(*X*|*B*∧*C*)^(*x*).*Prob*(*B*∧*C*) = *Prob*([*X *= *x*]∧*B*∧*C*) = *Prob*(*C*|[*X *= *x*]∧*B*).*Prob*([*X *= *x*]∧*B*)

and *Prob*([*X *= *x*]∧*B*) = *Prob*([*X *= *x*]|*B*).*Prob *(*B*) which we denoted *Prob*([*X *= *x*]|*B*) = *P*^(*X*|*B*)^(*x*) in the main text. It shows that *P*^(*X*|*B*∧*C*)^(*x*) is proportional to *P*^(*X*|*B*)^(*x*) provided *Prob*(*B*∧*C*) does not vanish, which is obviously true since such a condition *B*∧*C *has been observed experimentally and data recorded that underlie the estimation of P^(*X*|*B*∧*C*)^.

Accordingly, *P*^(*X*|*B*∧*C*)^(*x*) vanishes as soon as *P*^(*X*|*B*)^(*x*) vanishes, demonstrating the claimed absolute continuity. The proof straightforwardly extends to the case where *X *takes continuous values in a metric space and *P*^(*X*|*B*∧*C*)^(*x*), *P*^(*X*|*B*)^(*x*) are distribution functions (i.e. densities).

### Kullback-Leibler divergence

At each genome location *n*, the probabilities $P_{n}^{\left(X|B\right)}$ and $P_{n}^{\left(X|B\wedge C\right)}$ are defined on the same space (the state space *χ *of the feature *X*). The *Kullback-Leibler distance *(which is rather termed a *divergence *because of its asymmetry) then can be defined as a relative entropy:

$$\begin{array}{c} \left(P_{n}^{\left(X|B\wedge C\right)}||P_{n}^{\left(X|B\right)})\equiv -S(P_{n}^{\left(X|B\wedge C\right)}|P_{n}^{\left(X|B\right)}\right)\\ =\sum_{x\in X}P_{n}^{\left(X|B\wedge C\right)}(x)\ln\left[P_{n}^{\left(X|B\wedge C\right)}(x)/P_{n}^{\left(x|B\right)}(x)\right]\geq 0 \end{array}$$

in the discrete case, where ∑_x ∈ *χ *_should be replaced by $\int_{x}dx$ in case of a continuous-valued feature. Considering the symmetrized counterpart *D*(*B*∧*C*|*B*) + *D*(*B*|*B*∧*C*) of the Kullback-Leibler divergence would yield a *bona fide *distance, but it could take infinite values (if for some value *x *of the feature *X *the probability *P*^(*X*|*B*∧*C*)^(*x*) vanishes whereas *P*^(*X*|*B*)^(*x*) does not). When it is well-defined, it satisfies:

$$\begin{array}{c} D_{n}^{\left(X\right)}(B\wedge C|B)+D_{n}^{X}(B|B\wedge C)=\sum_{x\in X}\left[P_{n}^{\left(X|B\wedge C\right)}(x)-P_{n}^{\left(X|B\right)}(x)\right]\ln\left[P_{n}^{\left(X|B\wedge C\right)}(x)/P_{n}^{\left(x|B\right)}(x)\right]\\ \approx \sum_{x\in X}\frac{{\left(P_{n}^{\left(X|B\wedge C\right)}(x)-P_{n}^{\left(X|B\right)}(x)\right)}^{2}}{P_{n}^{\left(X|B\right)}(x)} \end{array}$$

where the latter approximation holds when $P_{n}^{\left(X|B\wedge C\right)}$ and $P_{n}^{\left(X|B\right)}$ are close enough, and amounts to a weighted *L*_2 _distance. We favor the use of the plain Kullback-Leibler divergence since it is always well-defined, i.e. it takes only finite values because of the absolute continuity (see above) of the probability distribution $P_{n}^{\left(X|B\wedge C\right)}$ with respect to $P_{n}^{\left(X|B\right)}$; moreover, its asymmetry parallels the intrinsic asymmetry of the comparison it intends to quantify, owing to the hierarchical relationship between the conditionings *B*∧*C *and *B *involved respectively in $P_{n}^{\left(X|B\wedge C\right)}$ and $P_{n}^{\left(X|B\right)}$.

The rationale for considering the Kullback-Leibler divergence rather than a *L*_*p *_distance is to weight the elementary contributions of each value *x *of *X *to the distance between the probability distributions by the probability of this value *x*; the distributions could differ significantly in *x *without having a significant divergence provided the probability of observing this value is in any case negligible. In the same spirit, Renyi generalizations can also be considered, replacing *z *ln *z *by (*q*-1)^-1^*z*^*q*^, which will allow the contribution of the rare events in the distance to be weighted differentially. Let us denote

$$D_{n}^{\left(X\right)}(B\wedge C|B)=\left(P_{n}^{\left(X|B\wedge C\right)}||P_{n}^{\left(X|B\right)}\right)$$

Its minimal value 0 is observed if *C *adds no new information. It has no a priori maximum: there is no other solution of the constrained variational equation than the case of equality of the two distributions. A maximal value is reached when *B*∧*C *fully conditions *X*, namely $P_{n}^{\left(X|B\wedge C\right)}(x)=\delta_{x,x_{0}}$, and moreover $P_{n}^{\left(X|B\right)}$ reaches its maximal value in *x*_0_; in this case, $D_{n}^{\left(X\right)}(B\wedge C|B)=-\ln\left[P_{n}^{\left(X|B\right)}(x_{0})\right]\geq S\left(P_{n}^{\left(X|B\right)}\right)$, which is equivalent to considering the ratio of the divergence to the entropy of the distribution *P*_*n*_^(*X*|*B*)^. We thus consider the ratio $D_{n}^{\left(X\right)}(B\wedge C|B)/S\left(P_{n}^{\left(X|B\right)}\right)$. It is close to 0 when *C *is spurious, and it is larger than unity in the above-mentioned example $P_{n}^{\left(X|B\wedge C\right)}(x)=\delta_{x,x_{0}}$ where *C *adds an essential condition turning *X *into a deterministic variable.

### Transcriptome data

The transcriptome data used in this study to illustrate the concept of circumstantial context are part of a study investigating the effect of the delta isoform of the general transcription factor TAF6 in apoptosis induction and its relationship to the transcription factor p53 [8]. The microarray data are accessible from the Gene Expression Omnibus database  under accession number: GSE10795.

### Transcriptome data preprocessing

The median normalized relative signal intensities from the indicated transcriptome experiments, representing three biological replicates for either the B+ (p53+/+) or the B- (p53-/-) biological conditions [8], were transformed into probability landscapes as described in [5]. Here, as the scope of the demonstration is restricted, and also the number of the analyzed samples is very moderate, the following simplifications were introduced in the calculation of the probability landscapes:

(1) The resolution of the p-annotation is at probe- and not nucleotide-level, as this is the smallest common denominator between the different samples and higher resolution therefore has no bearing.

(2) As the data originate from the same transcriptome technology, and belong to a single series, we have omitted the calculation of the quality estimating $P_{P_{n}}$. Anyhow $P_{P_{n}}$ has no effect on the circumstantial context.

(3) Both biological conditions were treated independently and no global rescaling of the probability landscapes between the two biological conditions (B+, B-) was performed for reasons similar to those above. Rescaling in this particular case would have marginally impacted the Kullback-Leibler divergence by a constant.

(4) The estimated coefficient of variance associated with each signal was not taken into account as it affects only $P_{P_{n}}$.

It should be kept in mind that the analysis presented here serves only as a proof-of-principle for the circumstantial context analysis developed, and does not aspire to investigate the features of the analyzed data systematically to the full extent using the probability landscape concept. Furthermore, the analysis presented here is probe-centered and hence only approximately comparable to the data analysis in [8] which is gene-centered, and where the probe-to-gene correspondence has been established [11].

The initial raw signal values, the P-values, and the different divergence measures are all provided as additional files 1, 2, 3. Those are equally accessible through our website ( (follow ->"web sources" ->"supplementary materials").

## Competing interests

The authors declare that they have no competing interests.

## Authors' contributions

AL and AB have jointly investigated the mathematical, computational, and experimental aspects of the idea, initially proposed by AB, upon which this work is based. Both authors have written the manuscript together. Both authors have read and approved the final manuscript.

## Supplementary Material

Additional file 1 File provides the probe IDs, the associated raw signal estimates of the two times three biological replicates (C1, C2, C3) of the two biological conditions (B+, B-), the probability profiles, and the Kullback-Leibler divergence estimates for the transcriptome data described in [8]. This first file contains the entire dataset of 31710 probes analyzed. Click here for file

Additional file 2 File provides the probe IDs, the associated raw signal estimates of the two times three biological replicates (C1, C2, C3) of the two biological conditions (B+, B-), the probability profiles, and the Kullback-Leibler divergence estimates for the 899 selected p53 modulated probes [8]. Click here for file

Additional file 3 File provides the probe IDs, the associated raw signal estimates of the two times three biological replicates (C1, C2, C3) of the two biological conditions (B+, B-), the probability profiles, and the Kullback-Leibler divergence estimates where the above mentioned 899 p53 modulated probes were swapped between the two biological conditions. Click here for file
